# Crosslinked flagella as a stabilized vaccine adjuvant scaffold

**DOI:** 10.1186/s12896-019-0545-3

**Published:** 2019-07-18

**Authors:** Casey M. Gries, Rohith R. Mohan, Dimitrios Morikis, David D. Lo

**Affiliations:** 1 Division of Biomedical Sciences, School of Medicine, University of California, Riverside, USA; 2 Department of Bioengineering, University of California, Riverside, USA

**Keywords:** Vaccine, Polymer, Innate immunity

## Abstract

**Background:**

Engineered vaccine proteins incorporating both antigen and adjuvant components are constructed with the aim of combining functions to induce effective protective immunity. Bacterial flagellin is a strong candidate for an engineered vaccine scaffold as it is known to provide adjuvant activity through its TLR5 and inflammasome activation. Moreover, polymerized flagellin filaments can elicit a more robust immunoglobulin response than monomeric flagellin, and the multimeric antigen form can also promote T cell-independent antibody responses. Here, we aim to produce and test a covalently stabilized polymerized flagellar filament, providing additional immune efficacy through stabilization of its polymeric filament structure, as well as stabilization for long-term storage.

**Results:**

Computational modeling of monomer packing in flagellin filaments helped identify amino acids with proximity to neighboring flagella protofilaments. Paired cysteine substitutions were made at amino acids predicted to form inter-monomer disulfide cross-links, and these substitutions were capable of forming flagella when transfected into a flagellin-negative strain of *Salmonella enterica* subspecies Typhimurium. Interestingly, each paired substitution stabilized different helical conformational polymorphisms; the stabilized filaments lost the ability to transition between conformations, reducing bacterial motility. More importantly, the paired substitutions enabled extensive disulfide cross links and intra-filament multimer formation, and in one of the three variants, permitted filament stability in high acidic and temperature conditions where wild-type filaments would normally rapidly depolymerize. In addition, with regard to potential adjuvant activity, all crosslinked flagella filaments were able to induce wild-type levels of epithelial NF-κB in a cell reporter system. Finally, bacterial virulence was unimpaired in epithelial adherence and invasion, and the cysteine substitutions also appeared to increase bacterial resistance to oxidizing and reducing conditions.

**Conclusions:**

We identified amino acid pairs, with cysteine substitutions, were able to form intermolecular disulfide bonds that stabilized the resulting flagellar filaments in detergent, hydrochloric acid, and high temperatures while retaining its immunostimulatory function. Flagellar filaments with disulfide-stabilized protofilaments introduce new possibilities for the application of flagella as a vaccine adjuvant. Specifically, increased stability and heat tolerance permits long-term storage in a range of temperature environments, as well as delivery under a range of clinical conditions.

**Electronic supplementary material:**

The online version of this article (10.1186/s12896-019-0545-3) contains supplementary material, which is available to authorized users.

## Background

Current vaccines and their adjuvants are restricted by a limited local and systemic immune induction and insufficient molecular stability due to a dependence on the storage and shipment “cold chain”. In 2012 the World Health Organization proposed new programmatic suitability requirements and preferred characteristics for vaccines; ideally one that is high-efficacy, multi-antigen, and heat-stable to be stored for extended periods of time above 8 °C with the current target threshold temperature of 40 °C [1]. Indeed, the use of biosynthetic vaccines and adjuvants has risen from inactivated whole components due to increasing safety demands and better-defined vaccine elements. However, this has necessitated the inclusion of appropriate adjuvants to enhance the immune responses induced by engineered antigens and is of particular importance when used for individuals suffering from immunodeficiency disorders.

Flagellin is a highly-conserved bacterial protein required for motility and adhesion to host tissues that elicits strong inflammatory responses in both plants and animals. The flagellin filament (flagella) is composed of thousands of individual flagellin proteins polymerized into 11 protofilaments, linked by non-covalent bonds, to form whip-like helical appendages hundreds of nanometers long and anchored at one end to a membrane-bound motor assembly. The non-covalent nature of the flagella allows for flexibility and conformation polymorphism. The flagellin monomer is divided into four domains: two terminal; D0 and D1, and two central; D2 and D3. The terminal, alpha-helical, regions are highly conserved between bacterial species and indispensable for filament polymerization as they are involved in the protein-protein interactions. The D1 domain contains the small but highly conserved region of flagellin primarily involved in immune recognition. The central regions, dispensable for flagella polymerization and function, are exposed on the filament and are highly variable. These regions define the various H-antigen subtypes and allow for the evasion of adaptive immune responses [2].

Innate immune pattern recognition receptors (PRRs), including toll-like receptors (TLRs) and NOD-like receptors (NLRs) recognize pathogen-associated molecular patterns (PAMPs) and binding of these ligands to their cognate receptors initiates a signaling cascade that results in the activation and maturation of antigen-presenting cells (APCs) which, in turn, activate adaptive immune responses. Flagellin is the sole agonist of TLR5 [2] which mobilizes NF-κB via a MyD88-dependent pathway. Intracellular flagellin is recognized by two NLR family receptors, NLRC4 and NAIP5, which activate the inflammasome. As opposed to many other PAMPs, flagellin produces a mixed Th1 and Th2 immune response [3, 4], considered important to mounting an effective immune memory response. However, the immune recognition site of flagellin is hidden, buried in the flagella core [2], suggesting that flagellin can be recognized by PRRs only in its monomeric form.

Interestingly, several studies have demonstrated that polymeric flagellin has equal if not greater immunostimulatory effects as a vaccine adjuvant compared to monomeric flagellin. We previously employed a hybrid engineered flagellin substituting the central flexible domain with the Env protein from Dengue Virus [5]. The resulting hybrid flagellin was found to be a more potent vaccine in polymeric versus monomeric form. This effect was due in part to the ability of the filament to efficiently crosslink B cell receptors; moreover, these filaments were also able to induce a T helper cell independent immune response, adding T helper-independent B cells to the available antibody repertoire. In this study, our approach is to further stabilize the flagellar filament scaffold by introducing specific cysteine amino acid substitutions in adjacent protofilament monomers to introduce intermolecular disulfide bonds.

## Results

### Introducing intermolecular disulfide bonds in FliC filaments

The flagella of *Salmonella* (In this study, we used *Salmonella enterica* serovar Typhimurium, referred to briefly as *S.* Typhimurium in this manuscript) consist of a filament that is a supercoiled assembly of monomers of proteins such as flagellin (FliC). The monomers have a shape resembling the capital Greek letter gamma (Γ), with the tail of the monomer consisting of alpha-helical domains joined to the arm by a flexible central domain. The alpha-helical domains align to form protofilaments. Eleven protofilaments form the flagellar filament, through translation-rotation screw symmetry, with the flexible arm of each monomer presented to the external solvent-facing surface. No covalent links provide stability to the filament, so that filaments can bend or de-polymerize under certain conditions. Computational models of the packing of flagellin monomers within polymerized filaments allowed us to identify candidate amino acid pairs where neighboring monomers are capable of forming intermolecular disulfide bonds when replaced with cysteine.

**Fig. 1:**
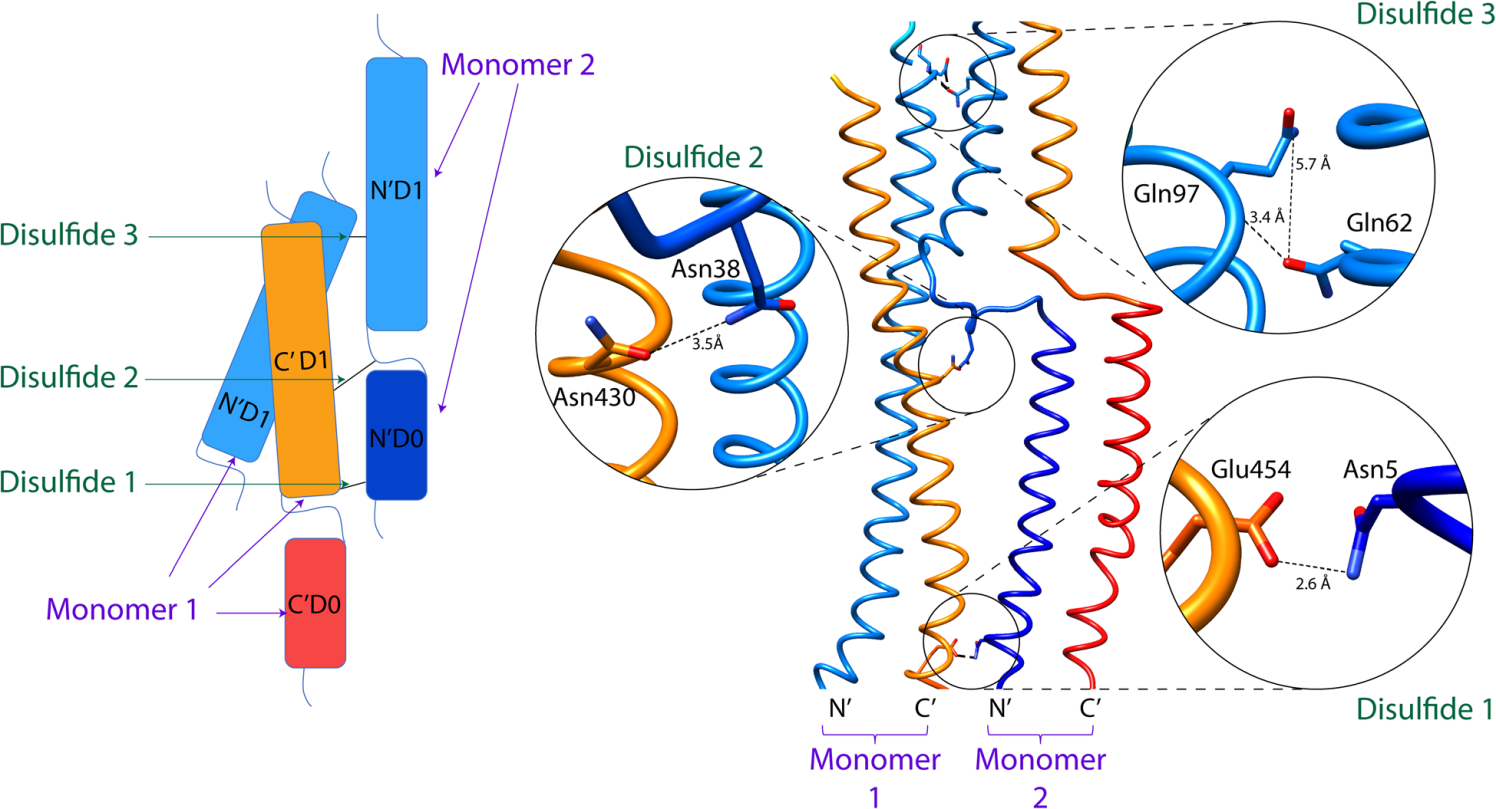
Introducing intermolecular disulfide bonds in FliC protofilaments. (Left) Depiction of the helical translations and proposed locations to introduce disulfide bonds between neighboring flagellin protofilaments. Domains N’D0 and N’D1 are colored dark and light blue respectively while C’D0 and C’D1 are orange and red, respectively. (Right) Molecular graphic representation of sites of interest within neighboring protofilament monomers. Insets show a zoomed and rotated view of the amino acid interactions with the specific distances shown: 1) Glu454[Oε1] – Asn5[Nδ1], 2) Asn430[Oδ1] – Asn38[Nδ1], and 3) Gln97[N] – Gln62[Oε1], Gln97[Nε2] – Gln62[Oε1]

The illustration in Fig. 1 depicts the D0-D1 domain interface of adjacent FliC protofilament monomers with the identified amino acids and their intermolecular distance indicated. Asparagine, glutamine, and glutamic acid residues were chosen because combinations of their rotameric states can assume the distance needed for the formation of the disulfide bridge upon replacement by cysteine. The interacting pairs of amino acids selected connect (see Fig. 1): Disulfide 1) the C’D1 domain of protofilament monomer 1 with the N’D0 domain of protofilament monomer 2; Disulfide 2) the C’D1 domain of protofilament monomer 1 with the N’D0-N’D1 linker of protofilament monomer 2; and Disulfide 3) the N’D1 domain of protofilament monomer 1 with the N’D1 domain of protofilament monomer 2. Incorporation of disulfide bridges through mutations to Cys at the aforementioned sites is expected to improve the stability of the multimeric complexes without significantly altering the local structures and leaving the D2 and antigenic D3 domain unrestrained. The domain D1 is more amphipathic (and more stable) than domain D0, thus the cross-link at the helical N-terminus and at the linker at the other end will preserve its flexibility. As has been suggested in previous studies, the D0 domain and lower parts of the D1 domain do not have a significant effect on the structure of the monomer [6]. Thus, we propose that the identified amino acid substitutions would permit stabilizing intermolecular disulfide bond formation, retain filament formation, and allow for TLR5 immune recognition. Moreover, these studies leave the antigenic D3 domain intact for future introduction of immunogenic vaccine antigens, including substitution of the D3 domain with an intact virus protein domain, as we have reported previously [5].

### Cysteine replacements produced stabilized protofilaments with characteristic alignments

**Fig. 2:**
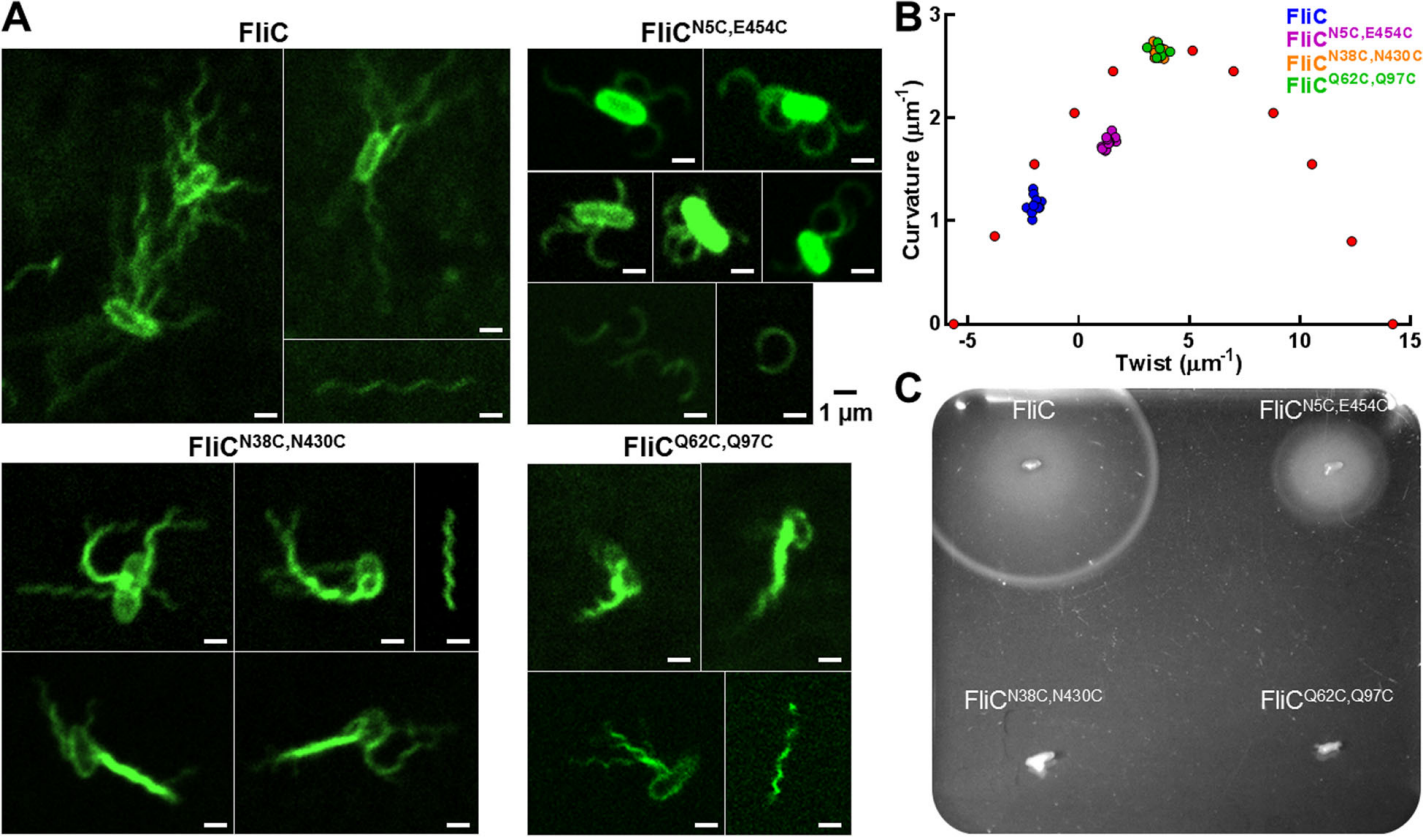
Assessing the structure and function of cysteine-substituted flagella. **a** Fluorescent staining of S. Typhimurium shows production and structural appearance of the respective flagella. Images included from three independent experiments. **b** Measurements of helical curvature (μM^− 1^) and twist (μM^− 1^) from each modified flagella compared to theoretical helical structures (red symbols). Multiple measurements were taken from both attached and detached filaments. Data (*n* ≥ 6) from two independent experiments. **c** Soft-agar motility assay depicting the effect of cysteine substitutions on the swimming motility of *S.* Typhimurium. Image representative of three independent experiments

Cysteine substitutions were performed in the three candidate pairs, generating FliC^N5C,E454C^, FliC^N38C,N430C^ and FliC^Q62C,Q97C^ (Additional file 2: Figure S1), and each was expressed *in trans* under control of the native *fliC* promoter in the *S.* Typhimurium Δ*fliC* strain SPN313 [7]. To ensure production and also assess the structure of flagella containing the point mutations, SPN313 cells harboring the above plasmids were stained with an Alexa Fluor 488-labelled carboxylic acid TFP ester dye and imaged using confocal microscopy. As shown in Fig. 2a, imaging of the stained filaments showed that for each of the paired cysteine replacements, characteristic filament wavelengths and curvatures were generated. Measurement of flagellar helical pitch and radius allowed for calculation of twist and curvature associated with known and theoretical filament waveforms based on the model that each protofilament exists in either left- or right-handed conformation that differs in length [8–14]. The ratio of left- and right-handed protofilaments (L:R) determined the overall filament helical shape. Compared to the “normal” (L:R of 2:9) appearing wild-type flagella, the FliC^N5C,E454C^ filament closely resembled a “coiled” (3:8) structure, while both FliC^N38C,N430C^ and FliC^Q62C,Q97C^ filaments resulted in “curly” (5:6) filaments (Fig. 2b), suggesting that either the cysteine substitution(s) or presence of disulfide bonds alter the composition of left- vs. right-handed protofilaments.

Stabilization of filaments in specific protofilament alignments might have consequences for bacterial mobility. With wild-type flagella, torsional stress due to flagellar rotation during bacterial motility is thought to be reflected in different protofilament alignments with corresponding filament wavelengths. A change in rotation of the filament causes a tumbling movement to enable changes in direction, and the change in torsional stress also causes changes in protofilament alignments. To determine whether the stabilization of the cysteine-substituted flagella in fixed protofilament alignments affected the function of the flagella, wild-type FliC, FliC^N5C,E454C^, FliC^N38C,N430C^ and FliC^Q62C,Q97C^ expressed *in trans* in the non-motile *S.* Typhimurium SPN313 was examined for their ability to restore swimming motility on semi-solid media. Soft agar motility assays revealed that, despite the production of full length flagella, SPN313 expressing FliC^N38C,N430C^ and FliC^Q62C,Q97C^ remained completely non-motile while expression of FliC^N5C,E454C^ restored approximately 50% of normal motility compared to wild-type FliC (Fig. 2c).

### Cysteine substitutions crosslink and stabilize FliC filaments

**Fig. 3:**
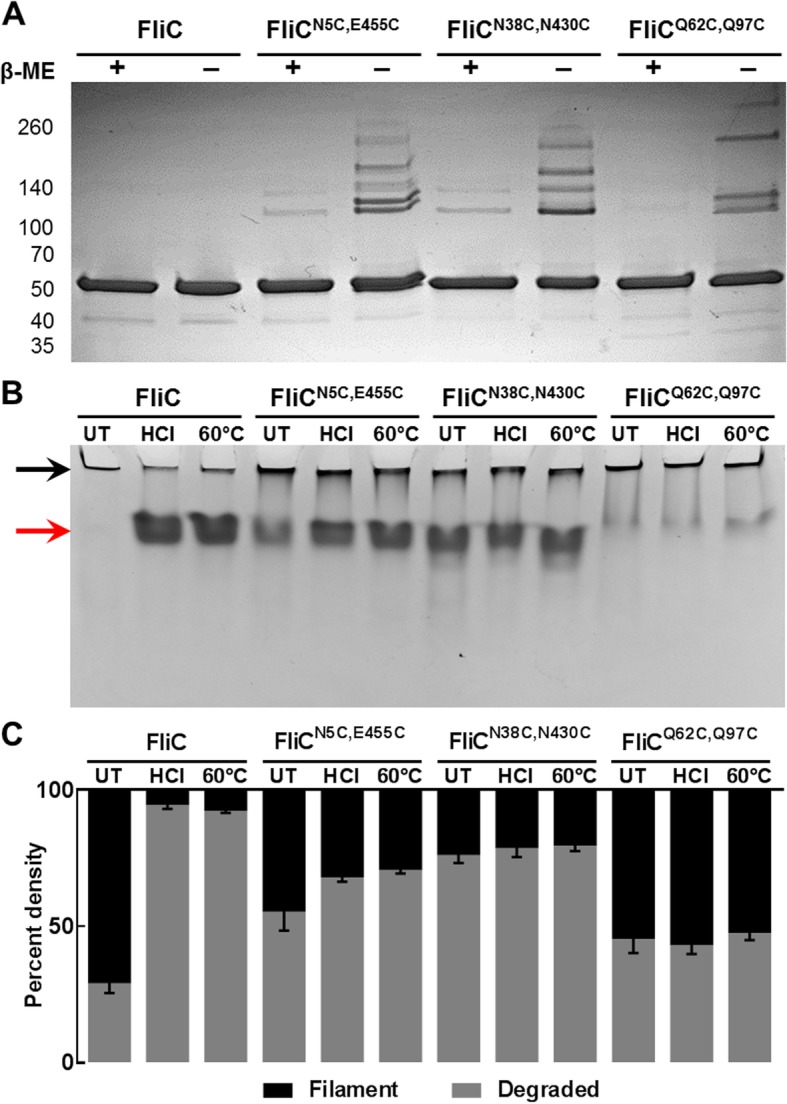
Crosslinking and stability of cysteine-substituted flagella. **a** Extracted filaments subjected to SDS-PAGE with and without β-mercaptoethanol (β-ME) reduction signifying the presence of disulfide bonds in all three cysteine-substituted flagella. Image representative of three independent experiments. **b** Thermal and acidic filament stability assessed by Native-PAGE following treatment with 50 mM HCl or heating at 60 °C for 10 min compared to untreated (UT). Image representative of two independent experiments. **c** Densitometry quantitation of replicate Native-PAGE gels as shown in **b**. Data included from two independent experiments with standard deviation shown

To assess protofilament crosslinking, extracted intact flagella from *S.* Typhimurium SPN313 expressing wild-type FliC, FliC^N5C,E454C^, FliC^N38C,N430C^ or FliC^Q62C,Q97C^ were analyzed using SDS-PAGE under reducing and non-reducing conditions (Fig. 3a). Consistent with crosslinked multimers, each of the paired cysteine replacements generated flagella retaining high-order oligomeric structures while the wild-type flagella ran solely as the 52 kDa monomer. Importantly, the polymers were reduced to monomeric form with the addition of β-mercaptoethanol (β-ME), signifying that the FliC^N5C,E454C^, FliC^N38C,N430C^ and FliC^Q62C,Q97C^ protofilaments were indeed crosslinked with disulfide bonds.

Traditional depolymerization of flagellar filaments is performed by lowering the pH with strong acids or incubating at temperatures 37 °C and above [15, 16]. To test the inherent thermal and acidic stability of the crosslinked filaments, extracted flagella were incubated in either 50 mM HCl or at 60 °C for 10 min. Native-PAGE analysis revealed that while wild-type FliC filaments were depolymerized following treatment with both HCl and 60 °C treatments, FliC^N5C,E454C^, FliC^N38C,N430C^ and FliC^Q62C,Q97C^ substitutions retained a mixture of both full-length filaments (black arrow) and depolymerized monomers (red arrow), with FliC^Q62C,Q97C^ completely unaffected by the treatments compared to untreated samples (Fig. 3b). Densitometry quantification of the respective filament and depolymerized protein bands from replicate Native-PAGE images is shown in Fig. 3c.

### Cysteine substitutions and bacterial viability

**Fig. 4:**
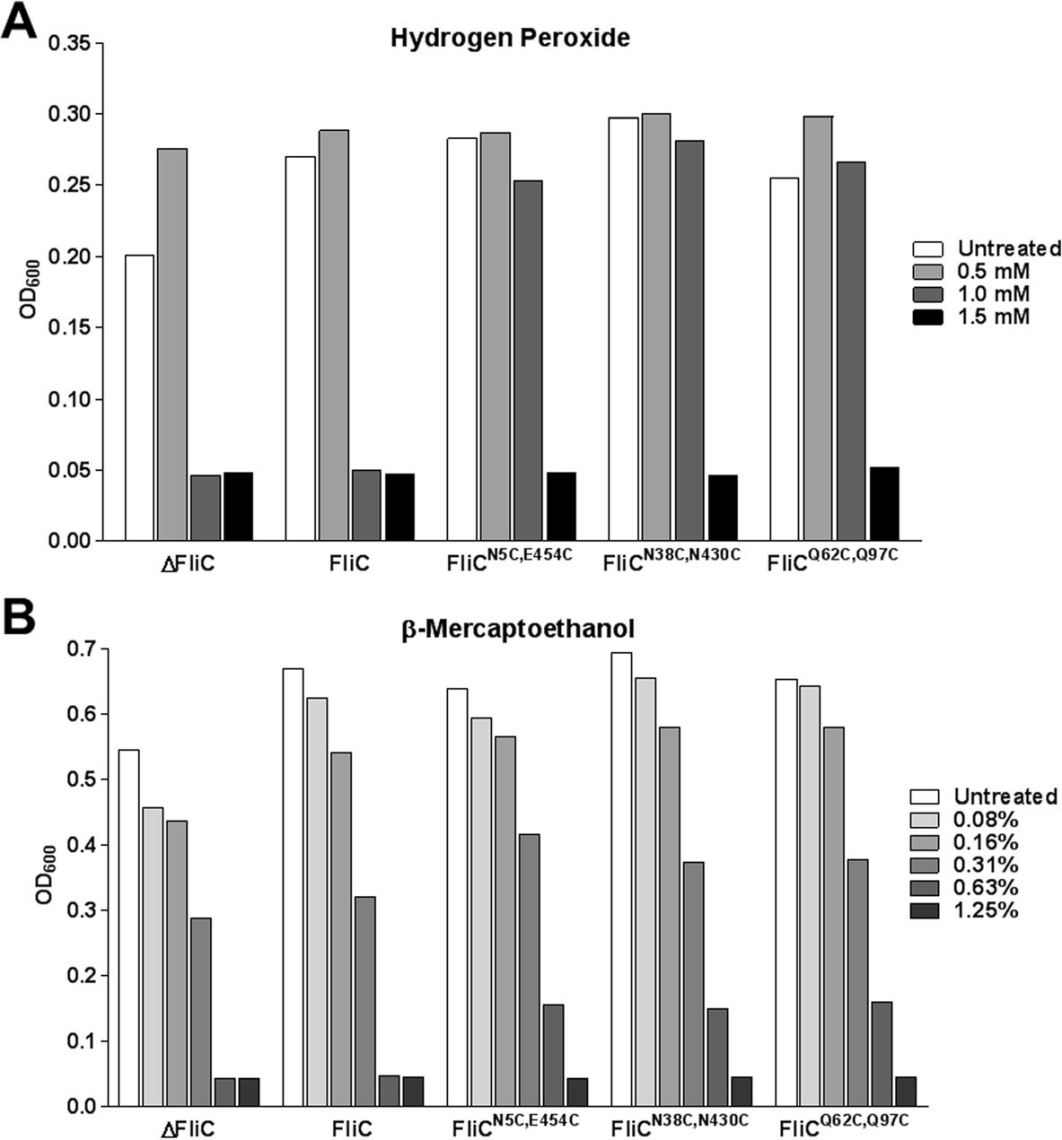
Oxidant and reductant buffering compacity of cysteine-substituted flagella. Growth of SPN313 in (**a**) hydrogen peroxide (**b**) β-mercaptoethanol using a broth microdilution method. Cultures harboring the indicated plasmids were incubated statically for 16 h at 35 °C, and OD_600_ was measured to determine the MIC preventing growth. ΔFliC represents *S.* Typhimurium SPN313 harboring the empty parent vector. Data representative of two independent experiments

While the cysteine substitutions enabled disulfide cross-links between monomers, the variable numbers of flagellin multimers observed using gel electrophoresis suggested that many of the available cysteines remained unconjugated. These cysteines might have not have been exposed to sufficiently oxidizing conditions to form disulfide bonds with neighboring cysteines, or they might simply not have been close enough to an available cysteine during filament formation. To examine the oxidizing and reducing capacity of SPN313 expressing wild-type FliC, FliC^N5C,E454C^, FliC^N38C,N430C^ and FliC^Q62C,Q97C^ a broth microdilution assay was performed using hydrogen peroxide (Fig. 4a) and β-mercaptoethanol (Fig. 4b) to assess their respective minimal inhibitory concentrations (MIC). Interestingly, when exposed to increasing concentrations of oxidizing (peroxide) or reducing (β-mercaptoethanol) conditions, the cysteine-substituted flagellins significantly enhanced bacterial viability. Thus, unbound cysteines in the flagella still appeared to have sufficient solvent exposure to provide capacity for hydrogen peroxide buffering in the surrounding environment. Alternatively, reduction of the crosslinked cysteines also allowed for enhanced survival in toxic conditions, together indicating that additional oxidation of the disulfide bonds is possible in downstream production and may provide enhanced filament stability.

### Crosslinked FliC filaments retain innate immune activation and epithelial invasion

**Fig. 5:**
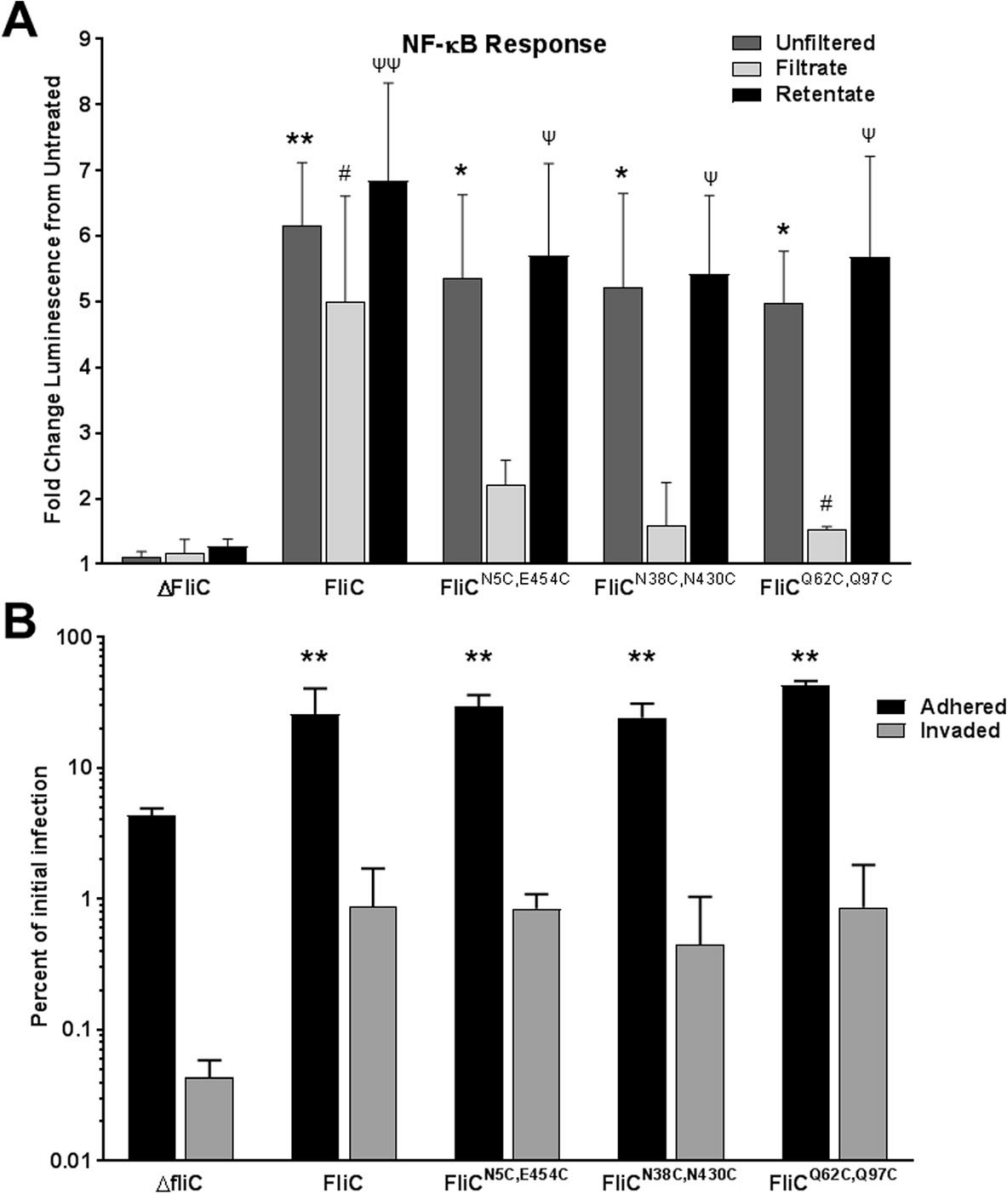
Immune response and functional effect of crosslinked flagella in Caco-2 cells. **a** Portions of extracted flagella were centrifuged though 100kdA filters to separate retentate filaments from filtrate monomers and, in addition to the original unfiltered extracts, 120 ng of each was added to Caco-2 epithelial monolayers harboring a NF-κB luciferase reporter vector. After 6 h, firefly and renilla luciferase were measured to assess the induction of NF-κB. Results include data from three independent experiments with standard error shown. Asterisk, hash, and psi symbols indicate statistically-significance difference comparing the unfiltered, filtrate, and retentate treatments, respectively, to ΔFliC. **b** Adherence and invasion assay performed with *S.* Typhimurium SPN313 cells harboring the empty vector (ΔFliC) or encoding the FliC derivatives were added to the apical side of Caco-2 cells. Invasion and adherence were assessed after 2 h and compared to the initial inoculum. Data from two independent experiments with standard deviation shown. Asterisk symbol indicates statistically-significance difference compared to ΔFliC. Single symbol; *p* < 0.05, double symbol; *p* < 0.01 as determined by 2-way ANOVA with Tukey’s multiple comparisons test

The flagellin monomer is known to trigger innate immune responses via the Toll-like receptor TLR5 and the inflammasome NLRC4. The linear peptide sequence recognized by TLR5 is within the alpha helical domain and would be expected to be hidden when monomers are contained within the polymerized flagellar filament. Stabilization of the filament by disulfide cross-linking might alter the availability of the TLR5 recognition sequence, thereby affecting the potency in activating innate immune responses. To test the ability of the crosslinked FliC variants to induce an immune response, Caco-2 intestinal epithelial cells transfected with an NF-κB luciferase reporter vector were treated with extracted flagellar filaments from *S.* Typhimurium SPN313. As shown in Fig. 5a, compared to extract from the empty plasmid, unfiltered FliC^N5C,E454C^, FliC^N38C,N430C^ and FliC^Q62C,Q97C^ induced NF-κB activity to a similar extent as WT FliC. To further asses the contributions of the individual fractions, flagella preparations were filtered to separate longer filaments from monomers and tested for relative potency in activating NF-κB responses. Interestingly, filaments retained by the filters proved be more potent stimulators of NF-κB (Fig. 5a).

To further examine the ability of the crosslinked flagella to function during in vivo-related processes, *S.* Typhimurium adherence and invasion assays were performed using Caco-2 epithelial cells (Fig. 5b). Compared to SPN313 containing the parent vector (ΔFliC), expression of all three crosslinked flagella variants enabled adherence and invasion of the epithelial cell layer equal to the expression wild-type FliC. Together, these data indicate that the crosslinked filaments retain their ability to function as normal flagella during host/immune processes.

### Long-term storage stability of crosslinked flagella at elevated temperatures

**Fig. 6:**
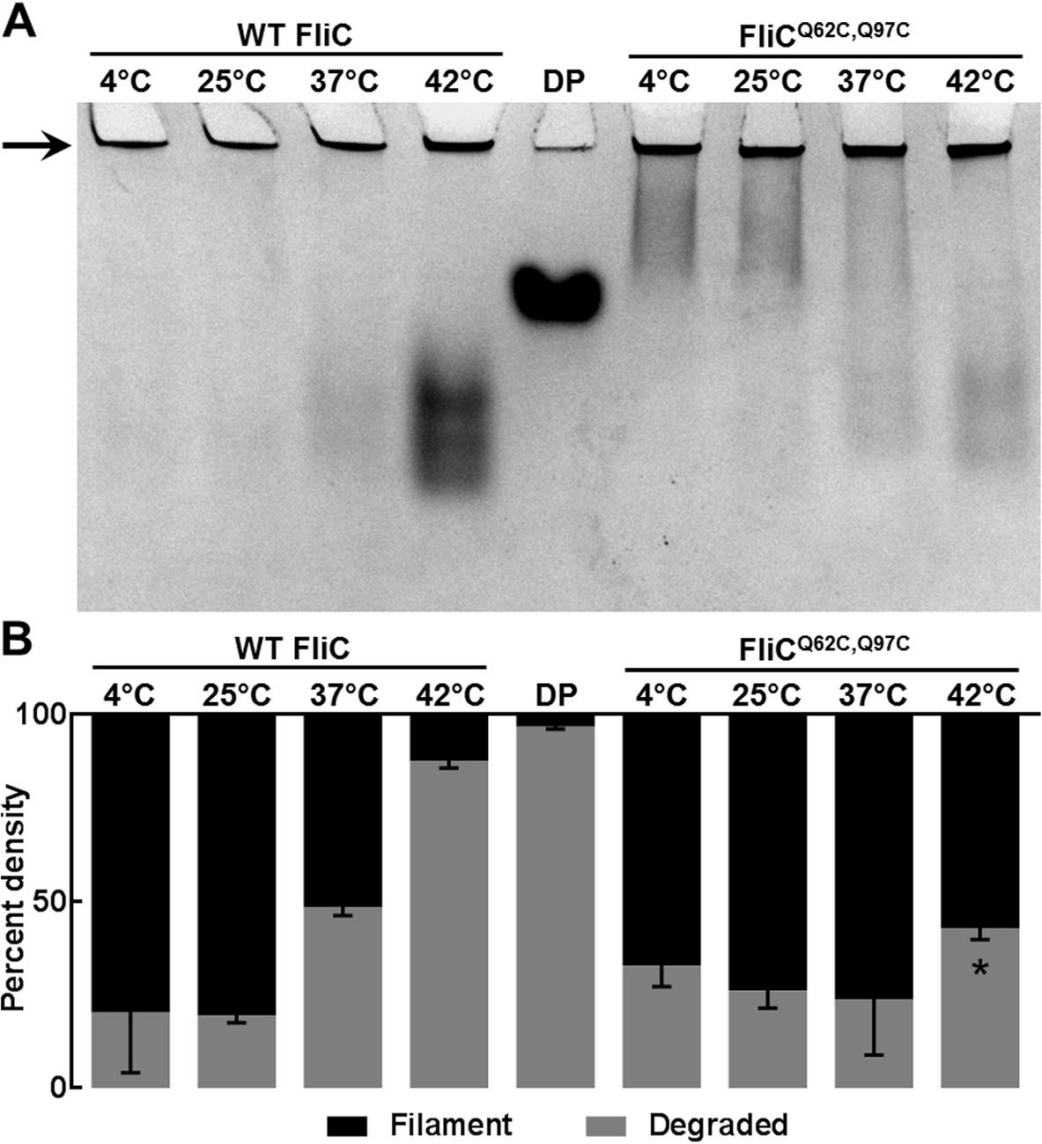
Long-term temperature stability of FliC^Q62C,Q97C^ in solution. **a** Solutions of extracted WT and FliC^Q62C,Q97C^ flagella filaments were incubated at 4 °C, 25 °C, 37 °C, and 42 °C for 7 days, and 3 ng was subjected to Native-PAGE to assess filament stability, depolymerization, and degradation. Image representative of two independent experiments. DP indicates an 80 °C depolymerized WT control sample. **b** Densitometry quantification of filaments (black arrow) and degraded / depolymerized protein. Data includes measurements from two independent experiments with standard deviation shown. Asterisk indicates statistically-significance (*p* < 0.05) difference compared to WT FliC as determined by 2-way ANOVA with Tukey’s multiple comparisons test

The FliC^Q62C,Q97C^ mutation appeared to confer significant thermal stability and resistance to depolymerization in acidic pH solutions, so we assessed the long-term stability of FliC^Q62C,Q97C^ filaments compared to wild-type FliC filaments in solution. Solutions of extracted flagella filaments were incubated for 7 days at 4 °C, 25 °C, 37 °C, and 42 °C, and filament survival was examined by Native-PAGE, including a depolymerized (DP) control sample. As shown in Fig. 6, a marked portion of WT FliC was depolymerized and degraded following incubation at 37 °C and 42 °C while that of FliC^Q62C,Q97C^ remained remarkably intact.

## Discussion

In our previous studies using an engineered hybrid flagellin, we replaced the D3 central domain with the coding sequence of the Dengue virus Env protein, and found that the monomer retained its ability to form polymeric filaments [5]. The filament proved to be more potent as an immunogen than the monomer, attributable in part to its potency as a polymeric antigen, but the protein might also have been protected from degradation by its assembly into the filaments. However, since flagella can depolymerize in aqueous solutions, we sought additional strategies to stabilize the filament structure that may provide resistance to depolymerization across a range of conditions including survival in vivo when administered as an engineered vaccine. The present study shows that a variety of amino acid substitutions based on computer modeling of the protofilament assembly can be used to produce filaments stabilized by disulfide cross links.

In these studies the D3 domain was intact, so the cysteine-replacement mutant proteins were normal size and transfection into FliC-negative *Salmonella* allowed production of flagella by the bacteria. As a result, we were able to gain additional insights into the structure and function of filaments produced by the cysteine replacement mutants. For example, replacement mutants were found to stabilize filaments into different protofilament alignments with distinct impacts on bacterial motility. Whether these conformations will have differential impacts on immune activation in vaccine formulations remains to be tested. In addition, the availability of thiol groups on exposed cysteine side chains appeared to provide microbial resistance to different redox conditions, and this may also have some impact on the stability of the filaments in vivo.

The most important practical result from these studies is that computer modeling of the protofilament packing provided useful predictions on which amino acids could be substituted with cysteine to enable disulfide cross-linking of flagellin monomers. The resulting replacement mutants were all capable of forming flagella, and disulfide cross links were readily demonstrated in all cases, providing increased stability of the filaments. It is not clear what factors influence the percentage of cysteines able to form disulfide bonds. Preliminary studies indicated that treatment of filaments with peroxide did not increase crosslinking, so it may be that the protofilament conformations associated with each replacement mutant keep many of the available cysteines too far away from nearby cysteines to form disulfide bonds.

In sum, we have shown that an engineered version of bacterial flagellin can be produced that provides increased stability through the introduction of disulfide crosslinks while retaining the general structural features of the flagellar filaments including innate immune agonist activity. This scaffold will be a useful starting point in the design of engineered synthetic vaccines that provide protein stability, adjuvant activity, and polymeric antigen presentation to provide more potent activation of both T helper-dependent and -independent B lymphocyte responses.

## Conclusions

Computer modeling of the polymerized flagellin filament identified nearby amino acid residues in adjacent monomers. Substituting cysteine residues at paired adjacent sites allowed us to test whether monomers could form covalent disulfide bonds in the flagellin filament. Bacteria expressing these cysteine-substituted flagellin sequences produced flagellae with a high proportion of disulfide cross-linked flagellin multimers. The filaments showed stabilized helical conformations as well as high thermal stability in aqueous solution. These engineered filaments show promise as a stable scaffold for polymeric vaccines.

## Methods

### Computational modeling

The program UCSF Chimera [17] was utilized for visualization, intermolecular interaction analysis, and mutation design. An R-type flagellar filament structure, made of flagellin monomers (PDB Code 1UCU [10]) was used for modeling. Guided by structural and physicochemical analysis, potential sites for cross-linking through the introduction of disulfide bridges, and thus stabilization of monomer-monomer interfaces, were sought at the flexible elongated domains D0 and D1, while leaving D2 and antigenic domain D3 unrestrained. Mutations of asparagine, glutamine, and glutamic acid residues to cysteines were introduced at critical positions in D0 and D1 domains (Fig. 1). Given the largely polar nature of inter-monomer interfaces, mutations involved sites of side chain interactions that contribute to the stability of the dimers only through weak hydrogen bonds. The rationale was to replace these weak, and easily breakable, non-bonded interactions with strong covalent disulfide bridges, and thus strengthen the stability of the dimers. Asparagine, glutamine, and glutamic acid residues were selected in mutation pairs, E454(A)-N5(B), N430(A)-N38(B), and Q97(A)-Q62(B), because combinations of their side chain rotameric states can assume the distance required for the formation of an inter-monomer disulfide bridge, upon replacement by cysteine. Finally, mutations were chosen at locations that would not potentially compromise the integrity of the helical structures of D0 and D1. Finally, each chosen disulfide bridge contains at least one cysteine at a (typically dynamically fraying) helical terminus (D0 or D1), or at a (typically dynamically mobile) loop (D0-D1), to assure necessary flexibility for the steric accommodation of the disulfide bridge.

### Bacterial strains and cloning techniques

The *fliC* gene and promoter elements were amplified from the *Salmonella enterica* serovar Typhimurium IR715 (“*S*. Typhimurium” throughout the text) chromosome using oligonucleotides fliC-F and fliC-R (Additional file 1: Table S1) which encode restriction sites BamHI and EcoRI used for cloning into the pENTR3C vector (Invitrogen, USA), generating pFliC. Oligonucleotides encoding desired polymorphisms were used to divergently amplify the entire plasmid. Resulting PCR products were treated with DpnI (New England Biolabs, USA) to remove template plasmid prior to transformation into *E. coli* DH5α. Plasmid sequences were confirmed at the UC Riverside IIGB core facility. All pFliC variants were transformed into the *S.* Typhimurium SPN313 (IR715 Δ*fliC fljB*::Mu*d*Cm) [7].

### Collection of flagellar filaments

Overnight cultures of *S.* Typhimurium SPN313 harboring FliC-encoding plasmids were diluted 1:100 in LB broth (Fisher, USA) containing 28 mM glucose and 50 μg/mL kanamycin and incubated for 16 h at 30 °C with 125 RPM aeration. Cultures were then centrifuged and cells washed with PBS. Cell suspensions were vortexed for 5 min and sheared flagellin were separated from the cells by centrifugation. Supernatants containing intact flagella were precipitated with acetone or pelleted by centrifugation for 1 h at 48,400 x g, 4 °C and resolubilized in PBS. Where required, flagellar filaments were enriched by centrifugation through 100,000 MWCO filters (Millipore, USA) and, when necessary, filaments were depolymerized at 80 °C for 10 min.

### Luminescence reporter assay

Caco-2BBe cells were cultured similarly to standard methods [18]. The SRE or NF-κB luciferase reporter vector (Qiagen, USA) was transfected into 5 × 10^4^ freshly harvested Caco-2 cells using Lipofectamine 3000 (Invitrogen). Following overnight incubation, transfected cells were treated for 6 h with 120 ng of flagellar protein. Firefly and renilla luminescence was detected using the Dual-Glo luciferase assay (Promega, USA). Three independent experiments were performed using multiple, independent flagellin collections.

### Flagella filament staining

Fluorescent imaging of *S.* Typhimurium flagella was performed similarly as previously described [19]. Briefly, late-logarithmic and early-stationary phase cultures of *S.* Typhimurium harboring FliC-encoding plasmids were washed with PBS and diluted to OD_600_ 0.2 in 0.1 mL Alexa Fluor 488 5-Tetrafluorophenyl (TFP) Ester (Invitrogen, USA) prepared according to manufacturer recommendations. Cells were incubated for 1 h in the dark at room temperature with occasional mixing and washed with PBS prior to imaging. Images were acquired on a CSU-X-1 spinning-disk confocal imager (Yokogawa, Japan) attached to a Zeiss Axio Observer inverted microscope. Hardware, including the Prim 95B digital camera (Photometrics, USA) was controlled by Micro-Manager imaging software. Three independent experiments were performed. Images were further optimized using ZEN software [Zeiss, Germany] and flagella measurements taken using ImageJ/Fiji software.

### Gel electrophoresis and densitometry

Native- and SDS-PAGE was performed using precast 4–12% mini-PROTEAN TGX gels (Bio-Rad, USA) with appropriate manufacturer-recommended sample and running buffers. Equal amounts of flagellin protein was loaded as measured by BCA (Thermo, USA) and protein molecular weights were determined using the Spectra BR ladder (Thermo, USA). Densitometry analysis was performed using ImageJ with individual histogram peaks representing the filament and all depolymerized and/or degraded products quantified separately. Each fraction is presented as a percentage of total area under the curve. The number of independent experiments performed are described in the figure legends.

### Broth microdilutions for MIC quantification

Overnight cultures of *S.* Typhimurium SPN313 harboring FliC-encoding plasmids were diluted to OD_600_ = 0.01 in 0.1 mL of LB broth containing a range of hydrogen peroxide or β-mercaptoethanol concentrations within 96-well plates. Cultures were incubated statically for 16 h at 35 °C and absorbance at 600 nm was read to asses growth. Two independent experiments were performed.

### Adherence and invasion assay

After the Caco-2 monolayer reached full differentiation (21 days) the medium was exchanged to antibiotics-free DMEM for 24 h. Bacteria were cultured overnight in LB + Kan^50^ at 35 °C with 150 RPM aeration then diluted 1:100 in fresh LB + Kan^50^ and cultured the same way for 5 h. Bacteria were then pelleted at 5,000×g for 5 min, washed with PBS, and diluted to OD_600_ 0.1 in DMEM (for MOI of 10). 500 μL of the bacterial suspension was added to the apical side of Caco-2 monolayers and incubated for 1 h at 37 °C. For invasion assays: after 1 h of incubation, cells were washed 2x with PBS and treated for 1 h with 50 μg/mL gentamicin in fresh DMEM + HEPES. For adherence assays: the 1 h gentamicin treatment was omitted. In all assays: 2 h post-infection, cells were washed 2x with PBS and lysed with 1% Triton X-100 for 10 min. Lysates were serial diluted in PBS and plated on LB agar to enumerate CFUs. Two independent experiments were performed.

### Statistics

When appropriate, statistical analyses were performed. Significant differences between experimental groups were determined with two-way analysis of variance (ANOVA) followed by Tukey’s multiple-comparison test, within Prism 7 (GraphPad, La Jolla, CA). For all analyses, a *P* value of less than 0.05 was considered statistically significant.

## Additional files

Additional file 1: **Table S1.** Oligonucleotides and plasmids. (DOCX 16 kb)

Additional file 2: **Figure S1.** *Salmonella enterica* Typhimurium fliC. (PDF 164 kb)

## Data Availability

The datasets used and analyzed during the current study are available from the corresponding author on reasonable request.

## Abbreviations

ANOVA: Analysis of variance
APC: Antigen presenting cell
BCA: Bicinchoninic acid
CFU: Colony-forming units
MIC: Minimal inhibitory concentration
NF-κB: nuclear factor kappa B
NLR: NOD-like receptor
NLRC4: NLR family CARD domain-containing protein 4
PAGE: Polyacrylamide gel electrophoresis
PAMP: Pathogen-associated molecular pattern
PBS: Phosphate-buffered saline
PRR: Pattern recognition receptors
SDS: Sodium dodecyl sulfate
TFP: Tetrafluorophenyl
TLR: Toll-like receptor
TLR5: Toll-like receptor 5
WT: Wild type
β-ME: Beta mercaptoethanol
