# Supplementary material for: Crosslinked flagella as a stabilized vaccine adjuvant scaffold
Source: BMC Biotechnol. 2019 Jul 18;19:48. doi: 10.1186/s12896-019-0545-3 (PMC6637544; doi:10.1186/s12896-019-0545-3)
Supplement: Supplementary file 1 — Table S1. Oligonucleotides and plasmids. (DOCX 16 kb) [file 12896_2019_545_MOESM1_ESM.docx]

**Additional file 1: Table S1 – Oligonucleotides and plasmids**

| **Oligonucleotides** | | |
| --- | --- | --- |
| **Name** | **Sequence (5’→3’)** | **Source** |
| fliC-F | CGAGGATCCTAAAGTTCGAAATTCAGGTGCC | This work |
| fliC-R | CCCGAATTCTTAACGCAGTAAAGAGAGGACG | This work |
| Asn5Cys-F | TTTGTACAAACAGCCTGTCGCTGT | This work |
| Asn5Cys-R | GTACAAATGACTTGTGCCATGATCTTTTCC | This work |
| Asn38Cys-F | TCTGCAGCGCGAAAGACGAT | This work |
| Asn38Cys-R | CTGCAGATACGCAGACCGGA | This work |
| Gln62Cys-F | CTTGTGCTTCCCGTAACGCTAAC | This work |
| Gln62Cys-R | GCACAAGTCAGACCTTTGATGTTCG | This work |
| Gln97Cys-F | TTTGCTCTGCTAACAGCACCAACT | This work |
| Gln97Cys-R | GAGCAAACCGCCAGTTCACG | This work |
| Asn430Cys-F | AGTGTCGTTTCAACTCCGCTATTACCA | This work |
| Asn430Cys-R | CGACACTGTACCGCACCCAG | This work |
| Glu454Cys-F | TCTGCGATTCCGACTACGCGA | This work |
| Glu454Cys-R | TCGCAGATACGGCTACGGGC | This work |
| **Plasmids** | | |
| **Name** | **Properties** | **Source** |
| pENTR 3C | pUC origin, Kanamycin resistance | Invitrogen |
| pFliC | WT *fliC* in pENTR 3C | This work |
| pFliC^N5C,E454C^ | Created with Asn5Cys & Glu454Cys in pFliC | This work |
| pFliC^N38C,N430C^ | Created with Asn38Cys & Asn430Cys in pFliC | This work |
| pFliC^Q62C,Q97C^ | Created with Gln62Cys & Gln97Cys in pFliC | This work |
