# Supplementary material for: Crosslinked flagella as a stabilized vaccine adjuvant scaffold
Source: BMC Biotechnol. 2019 Jul 18;19:48. doi: 10.1186/s12896-019-0545-3 (PMC6637544; doi:10.1186/s12896-019-0545-3)
Supplement: Supplementary file 2 — Figure S1. Salmonella enterica Typhimurium fliC. (PDF 164 kb) [file 12896_2019_545_MOESM2_ESM.pdf]

Salmonella enterica Typhimurium *fliC*

ATGGCACAAGTCATT**AAT**ACAAACAGCCTGTCGCTGTTGACCCAGAATAACCTGAACAAATCCCAGTCCG
CTCTGGGCACCGCTATCGAGCGTCTGTCTTCCGGTCTGCGTATC**AAC**AGCGCGAAAGACGATGCGGCAGG
TCAGGCGATTGCTAACCGTTTTACCGCGAACATCAAAGGTCTGACT**CAG**GCTTCCCGTAACGCTAACGAC
GGTATCTCCATTGCGCAGACCACTGAAGGCGCGCTGAACGAAATCAACAACAACCTGCAGCGTGTGCGTG
AACTGGCGGTT**CAG**TCTGCTAACAGCACCAACTCCCAGTCTGACCTCGACTCCATCCAGGCTGAAATCAC
CCAGCGCCTGAACGAAATCGACCGTGTATCCGGCCAGACTCAGTTCAACGGCGTGAAAGTCCTGGCGCAG
GACAACACCCTGACCATCCAGGTTGGTGCCAACGACGGTGAAACTATCGATATCGATCTGAAGCAGATCA
ACTCTCAGACCCTGGGTCTGGATACGCTGAATGTGCAACAAAAATATAAGGTCAGCGATACGGCTGCAAC
TGTTACAGGATATGCCGATACTACGATTGCTTTAGACAATAGTACTTTTAAAGCCTCGGCTACTGGTCTT
GGTGGTACTGACCAGAAAATTGATGGCGATTTAAAATTTGATGATACGACTGGAAAATATTACGCCAAAG
TTACCGTTACGGGGGGAACTGGTAAAGATGGCTATTATGAAGTTTCCGTTGATAAGACGAACGGTGAGGT
GACTCTTGCTGGCGGTGCGACTTCCCCGCTTACAGGTGGACTACCTGCGACAGCAACTGAGGATGTGAAA
AATGTACAAGTTGCAAATGCTGATTTGACAGAGGCTAAAGCCGCATTGACAGCAGCAGGTGTTACCGGCA
CAGCATCTGTTGTTAAGATGTCTTATACTGATAATAACGGTAAAACTATTGATGGTGGTTTAGCAGTTAA
GGTAGGCGATGATTACTATTCTGCAACTCAAAATAAAGATGGTTCCATAAGTATTAATACTACGAAATAC
ACTGCAGATGACGGTACATCCAAAACTGCACTAAACAAACTGGGTGGCGCAGACGGCAAAACCGAAGTTG
TTTCTATTGGTGGTAAAACTTACGCTGCAAGTAAAGCCGAAGGTCACAACTTTAAAGCACAGCCTGATCT
GGCGGAAGCGGCTGCTACAACCACCGAAAACCCGCTGCAGAAAATTGATGCTGCTTTGGCACAGGTTGAC
ACGTTACGTTCTGACCTGGGTGCGGTACAG**AAC**CGTTTCAACTCCGCTATTACCAACCTGGGCAACACCG
TAAACAACCTGACTTCTGCCCGTAGCCGTATC**GAA**GATTCCGACTACGCGACCGAAGTTTCCAACATGTC
TCGCGCGCAGATTCTGCAGCAGGCCGGTACCTCCGTTCTGGCGCAGGCGAACCAGGTTCCGCAAAACGTC
CTCTCTTTACTGCGT

MAQVI**N**TNSLSLLTQNNLNKSQSALGTAIERLSSGLRI**N**SAKDDAAGQAIANRFTANIKGLT**Q**ASRNAND
GISIAQTTEGALNEINNNLQRVRELAV**Q**SANSTNSQSDLDSIQAEITQRLNEIDRVSGQTQFNGVKVLAQ
DNTLTIQVGANDGETIDIDLKQINSQTLGLDTLNVQQKYKVSDTAATVTGYADTTIALDNSTFKASATGL
GGTDQKIDGDLKFDDTTGKYYAKVTVTGGTGKDGYYEVSVDKTNGEVTLAGGATSPLTGGLPATATEDVK
NVQVANADLTEAKAALTAAGVTGTASVVKMSYTDNNGKTIDGGLAVKVGDDYYSATQNKDGSISINTTKY
TADDGTSKTALNKLGGADGKTEVVSIGGKTYAASKAEGHNFKAQPDLAEAAATTTENPLQKIDAALAQVD
TLRSDLGAVQ**N**RFNSAITNLGNTVNNLTSARSRI**E**DSDYATEVSNMSRAQILQQAGTSVLAQANQVPQNV
LSLLR

N5C (AAT→TGT)
N38C (AAC→TGC)
Q62C (CAG→TGT)
Q97C (CAG→TGC)
N430C (AAC→TGT)
E454C (GAA→TGC)
